# Proteome analysis of the macroscopically affected colonic mucosa of Crohn’s disease and intestinal tuberculosis

**DOI:** 10.1038/srep23162

**Published:** 2016-03-18

**Authors:** Lokesh A. Rukmangadachar, Govind K. Makharia, Asha Mishra, Prasenjit Das, Gururao Hariprasad, Alagiri Srinivasan, Siddhartha Datta Gupta, Vineet Ahuja, Subrat K. Acharya

**Affiliations:** 1 Department of Biophysics, All India Institute of Medical Sciences, New Delhi, India; 2 Department of Gastroenterology and Human Nutrition, All India Institute of Medical Sciences, New Delhi, India; 3 Department of Pathology, All India Institute of Medical Sciences, New Delhi, India

## Abstract

Differentiation between intestinal tuberculosis (ITB) and Crohn’s disease (CD) is challenging in geographical regions where both these diseases are prevalent. There is a need of biomarkers for differentiation between these two disorders. Colonic biopsies from inflamed mucosa of treatment-naive patients with ITB, CD and controls were used for analysis. Protein extracted from biopsies was digested with trypsin and resulting peptides were labeled with iTRAQ reagents. The peptides were subsequently analyzed using LC-MS/MS for identification and quantification. Gene ontology annotation for proteins was analyzed in PANTHER. Validation experiments were done for six differentially expressed proteins using immunohistochemistry. 533 proteins were identified and 241 proteins were quantified from 5 sets of iTRAQ experiments. While 63 were differentially expressed in colonic mucosa of patients with CD and ITB in at least one set of iTRAQ experiment, 11 proteins were differentially expressed in more than one set of experiments. Six proteins used for validation using immunohistochemistry in a larger cohort of patients; none of them however was differentially expressed in patients with ITB and CD. There are differentially expressed proteins in tissue proteome of CD and ITB. Further experiments are required using a larger cohort of homogeneous tissue samples.

Intestinal tuberculosis (ITB) and Crohn’s disease (CD) are chronic inflammatory diseases of the intestine^1^,^2^,^3^,^4^. The clinical, morphological and histological features of ITB and CD are so similar that it becomes difficult to differentiate between these two entities^1^,^2^,^3^,^4^. In geographical regions such as India where both ITB and CD are prevalent, differential diagnosis between the two is challenging^5^,^6^. The natural history of CD is quite different from that of ITB^5^,^6^. While ITB gets cured with appropriate anti-tuberculosis treatment, CD has a remitting/relapsing or persistent course and stays life-long usually. Furthermore, biologics, which have now become one of the important treatment modalities for CD, their use however can lead to flare not only the latent tuberculosis but also the disease if the mistaken diagnosis of CD is made in a patient having ITB^5^,^6^,^7^. Because of similarity in the clinical presentation, endoscopic appearance and histological characteristics between these two diseases, many of them are treated empirically with anti-tuberculous drugs at times^2^,^3^,^4^,^5^,^6^,^8^,^9^,^10^,^11^,^12^. While anti-saccharomyces cerevisiae antibodies has been reported more often in patients with CD in comparison to that in ulcerative colitis; ASCA is also positive in almost half of patients with ITB and hence a differentiation between CD and ITB can not be made using ASCA^13^,^14^. There is a need for a biomarker which can differentiation CD from ITB, none really exist at present.

Keeping in view of the recent developments in the field of clinical proteomics, we used this technique to identify biological marker(s) that can differentiate ITB from CD. Proteomics is a promising technology and it is used to understand the biological processes involved in inflammatory bowel diseases (IBD)^15^. Since, both ITB and CD manifest first at the intestinal mucosa, comparing the mucosal proteome of these two diseases might help in identifying differentially expressed proteins, their possible networks and interactions involved in development of these two diseases. A proteomic approach has been used earlier for finding out differences between different forms of colitides and for exploring a biomarker for the activity of the disease^16^,^17^,^18^,^19^,^20^. Proteomics has also been employed in the area of tuberculosis research in the quest for biomarkers^21^. This technology has yet not been explored to detect potentially useful markers that can help differentiating ITB from CD.

In this study, we analyzed and compared the proteome of the inflamed colonic mucosa of patients with ITB and CD. Isobaric Tags for Relative and Absolute Quantification (iTRAQ) labeling technology followed by two-dimensional liquid chromatography and tandem mass spectrometry was used to compare the proteome of these two conditions for identification of potential biomarkers for differentiation of these two diseases with each other.

## Patients and Methods

Patients with CD and ITB were recruited from the Gastroenterology Clinic of All India Institute of Medical Sciences, New Delhi. The Ethics Committee of our Institution approved this study. The study was conducted as per guidelines of Indian Council of Medical Research/Good Clinical Practice. Informed and written consent was obtained from each participant of this study.

Complete evaluation of patients including their clinical, endoscopic, radiological and histological characteristics was done. Details of intake of anti-tuberculosis treatment in the past were documented. All patients underwent a colonoscopic (and retrograde ileoscopy where feasible) examination using a video-colonoscope after colon preparation with colonic lavage solution (polyethylene glycol). While as many as 30 patients were recruited and their biopsies were preserved in −80 °C, for the exploratory analysis of the proteome, the biopsies of only those patients were included where the follow up was complete and the diagnosis of either ITB or CD was confirmed. Since the present study was a pilot exploratory study, we have included 15 patients in the exploratory phase and 52 patients in the validation phase.

### Collection of biological samples

During colonoscopy, a segment-wise involvement of the colon was documented. Multiple mucosal biopsies, including five to six pieces from the macroscopically abnormal area (ulcerations, nodularity) and two to three biopsy pieces from the endoscopically normal looking areas, were obtained. Four bits of biopsies were fixed separately in 10% buffered formal-saline for histological features. After adequate fixation paraffin blocks were processed. From each block, 20 step sections of 4 μm thickness were prepared for detailed morphological assessment. The sections were stained by hematoxylin and eosin stain and were examined by two pathologists with special interest in gastrointestinal pathology who were blinded about the clinical data and final diagnoses of the patients. For proteomic study, 6–8 pieces of mucosal biopsies were obtained from the most involved area of the colon, mostly right side of the colon and ileocecal valve. Biopsies from the normal appearing colonic mucosa from ITB patients were also taken, which served as controls. These biopsies were snap frozen in liquid nitrogen and stored at −80 °C.

### Diagnostic criteria for CD and ITB

The diagnosis of CD was established on the basis of the European Crohn’s and Colitis Organization guidelines which is a combination of clinical, endoscopic and histological features^22^. The diagnosis of ITB was made on the basis of characteristic clinical features (abdominal pain, constipation and/or diarrhea, constitutional symptoms, and intestinal obstruction), endoscopic features (ileocecal area involvement, ulcerations, nodularity, and strictures), histological features (presence of granulomas), microbiological tests (presence of acid-fast bacilli on the smear examination or demonstration of acid fast bacilli by polymerase chain reaction), and response to anti-tuberculous treatment (Paustian’s criteria with Logan’s modification)^23^,^24^.

### Sample preparation for iTRAQ labeling and strong cation exchange chromatography

Snap frozen colonic biopsies were homogenized, re-suspended in lysis solution (8M urea, 2M thiourea and 4% CHAPS (3-[(3-Cholamidopropyl) dimethylammonio]-1-propanesulfonate]) and centrifuged at 10,000 g for 15 minutes at 4 °C. Debris was discarded and the supernatant was transferred onto a fresh Eppendorf tube. Protein extracted with lysis solution was buffer exchanged with 250 mM TEAB (Triethylammonium bicarbonate) using a 3 kDa cut off membrane filters to bring down the concentration of urea well below 0.1 M. Protein amount was quantified using the Bradford assay. 100 μg of protein was then trypsinized and labeled with 4 plex iTRAQ reagents (Siex, Framingham, MA, USA). Briefly, one unit of iTRAQ reagent required to label 100 μg of protein was thawed and reconstituted in 70 μl ethanol. Protein from each group was reduced, cysteine blocked and digested with trypsin. Tryptic peptides of reference pool which was pool of all 15 samples, CD, ITB and control groups were labeled with 114, 115, 116, and 117 iTRAQ tags, respectively, by incubating at room temperature for one hour for the first set of experiments. Remaining sets were labeled as given in the Table 1 on study design. The peptide mixtures belonging to each set were then pooled and dried in a speed vac. The pooled mixtures for each sample set were fractionated by a strong cation exchange chromatography.

Cation exchange chromatography was done using *liquid chromatography* system *(Shimadzu, Kyoto, Japan).* The peptide mixture was diluted with 1ml of buffer A (10 mM potassium phosphate, 25% acetonitrile, pH 2.9) and loaded onto a 2.1 mm × 150 mm cation column containing 5 μm particles and a 300 μm pore size *(ZORBAX SCX* 300; *Agilent Technologies,* Santa Clara, CA, USA). The peptides were eluted at a flow rate of 300 μl/minute with a gradient of 0% Buffer B (1M KCl, 10mM potassium phosphate, 25% acetonitrile, pH 2.9) for 10 minutes, 0–30% Buffer B for 25 minutes, 30–60% Buffer B for 10 minutes, 60–100% Buffer B for 5 minutes. The system was then maintained in 100% Buffer B for 10 minutes before equilibrating with 0% Buffer B for 30 minutes prior to the next injection. Thirty fractions were collected every minute. Monitoring of the elutions was dome by measuring absorbance at 220 nm, and vacuum-dried.

### Protein identification and quantitation

The peptides from each cation exchange fraction were resuspended in 0.1% formic acid and 3% acetonitrile solution and injected to a nano-LC system (*Tempo*^*TM*^; Siex, Framingham, MA, USA) equipped with a C18-75 μm × 150 mm column (*Michrom Bioresources, Inc. USA*). The samples were desalted online and an 84 minutes gradient of increasing acetonitrile concentration was used to separate the peptides. The eluent was sprayed into a tandem mass spectrometer (*QSTAR XL*; Siex, Framingham, MA, USA) and analyzed in Information Dependent Acquisition (IDA) mode using software (*Analyst QS 1.1*; Siex, Framingham, MA, USA). The collision energy was set with an intercept value +4 higher than that normally used for peptides, to provide for sufficient peptide fragmentation and generation of the iTRAQ reporter groups.

*Mascot*, version 3.2.2b, (*Matrix Sciences, London, UK*) was used for protein identification and quantitation from MS/MS data. For *Mascot* search, the nano-LC-ESI MS/MS data for 2+, 3+ and 4+ charged precursor ions were converted to centroid data, without smoothing, using the mascot.dll script in *Analyst QS1.1*. The MS/MS settings included: spectra de-isotoped except for the iTRAQ reporter region, peak areas reported, spectra rejected if they contained less than 10 peaks, and peaks not removed if they were close to the precursor m/z. The data were searched with a tolerance of 100 ppm for the precursor ions and 0.3 Da for the fragment ions. The following settings were used: trypsin was the cleavage enzyme, one missed cleavage allowed, carboxymethyl modification of cysteines was fixed modification and methionine oxidation was selected as a variable modification. Quantitaion method was iTRAQ 4 plex. Spectra were searched against *SwissProt* 57.15 database (515203 sequences; 181334896 residues) with taxonomy: *Homo sapiens* (human) (20266 sequences). The following settings were used to obtain the quantification results: the protein ratio type was the ‘weighted’ geometric mean, normalization with summed intensities and outlier removal was ‘automatic’. The peptide threshold was ‘at least homology’ (peptide score does not exceed absolute threshold but is an outlier from the quasi-normal distribution of random scores), the minimum number of peptides was two and peptides were required to be the top ranking peptide matches. An automatic decoy database search was also performed to estimate false discovery rate (FDR) for the search. The list of proteins identified and quantitated from each set was imported to an excel sheet. Since hemoglobin was consistently identified in each set of experiment, we normalized the data to one of the house keeping genes, beta actin, to exclude the effect of blood derived proteins. Average quantitation value for each protein was calculated and a t-test was performed for each protein, when it was present in more than two set of experiments.

### Bioinformatics analyses

Swiss Prot accession numbers of the proteins identified were used to extract gene ontology annotations from Protein Analysis through Evolutionary Relationships (PANTHER) classification system. Protein function and sub-cellular localization analyses were then performed using the gene ontology information and charts were made using the PANTHER (http://pantherdb.org; version 9.0) classification system. The PANTHER database was ideal for high throughput functional analysis for the datasets of protein sequences identified.

### Immunohistochemistry

Sections were cut from the archived paraffin blocks of colonic biopsies, dewaxed and rehydrated. Then endogenous peroxidase activity was blocked by 3% hydrogen peroxide (H_2_O_2_) and 0.1% protease, digested for 2 minutes at room temperature. The sections were then incubated with primary antibodies (Abcam, UK) and incubated overnight at 4 °C. Primary antibodies against Trefoil factor-3 (TFF3; 1:200), Thioredoxin (TRX; 1:500), Transgelin (SM22; 1:200), Tropomyosin (TPM; 1:50), IgGFc-binding protein (FCGBP; 1:200) and Myosin (MYH; 1:100) were used to validate the results of proteomic analysis as these proteins were found to be differently expressed in the inflamed mucosa of patients with CD and ITB.

The slides were then washed thrice with tris-buffered saline (pH 7.6) and finally incubated for 30 minutes with universal secondary antibody (BioCare Universal Kit, MACH4, Concord, CA, USA). The antigen-antibody reaction was visualized with peroxidase-substrate reaction by using 3, 3′-Diaminobenzidine (DAB) as chromogen. During interpretation a semi-quantitative grading as performed by considering total areas of stain expression and intensity of staining. The extent of protein staining was scored from 1 to 4, and the intensity of staining was scored from 1 to 3. The scores were then multiplied together and the final scores were classified as follows: 1–3, weak staining; 4–8, moderate staining; and 9–12, strong staining. A Fisher’s exact test was performed to assess the distribution pattern of the immunochemical score for expression of the protein.

## Results

### Proteomic experiments

**Figure 1:**
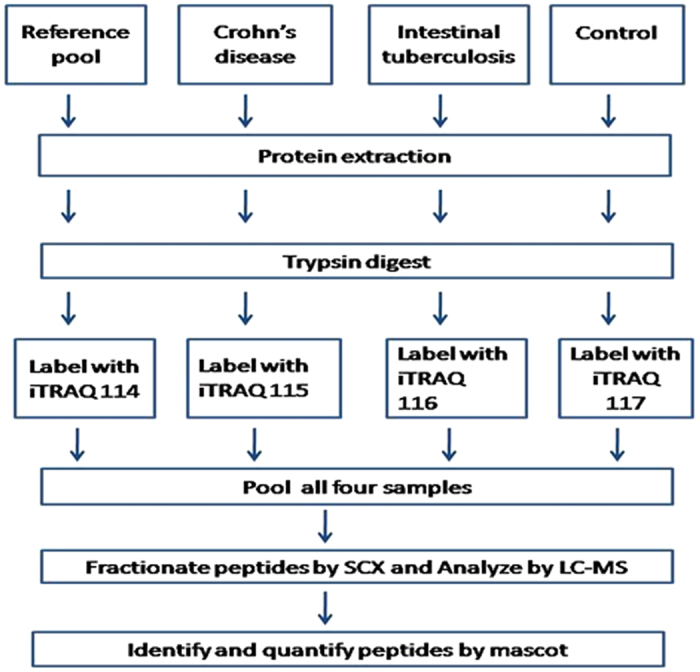
Biomarker discovery strategy adopted in iTRAQ method. The flow chart illustrates steps involved in the Set 1 to 5 of the iTRAQ analysis.

**Figure 2:**
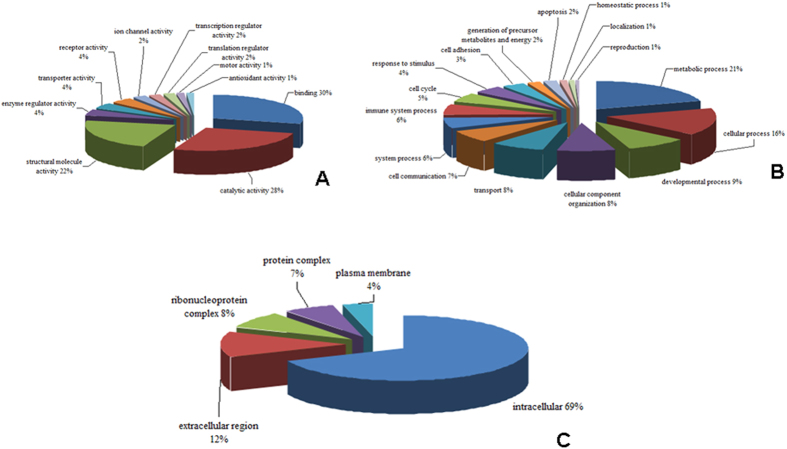
(**A**) Pie chart showing the Gene ontology annotation distribution of all the proteins identified in the macroscopically affected colonic mucosal biopsies in the proteome analysis. The proteins are grouped according to their molecular function. (**B)** Pie chart showing the Gene ontology annotation distribution of all the proteins identified in the macroscopically affected colonic mucosa in the proteome analysis. The proteins are grouped according to the known biological processes they are involved in. (**C**). Pie chart showing the Gene ontology annotation distribution of all the proteins identified in the macroscopically affected colonic mucosa in the proteome analysis. The proteins are grouped according to the cellular distribution.

The strategy used for the proteomics analysis in this study is shown in Fig. 1. The colonic mucosal biopsy samples from 15 patients (5 with ITB, CD and controls each) were used as per the design shown in Table 1. A total of 533 proteins were identified in the five sets of experiments carried out and 241 proteins among these were quantified with the criteria of at least two peptides being quantified in the mascot. One hundred and thirty eight proteins among these were identified and quantified in at least two sets of experiments (Table 2). The results from the five sets of experiments were comparable in terms of total number of proteins identified and quantified and false discovery rate was consistently low. It was noted that hemoglobin was consistently common. A Bioinformatics analysis of all the identified proteins was carried out in PANTHER. A detailed gene ontology analysis of these proteins has been depicted in Fig. 2.

### Differential expression of proteins in the macroscopically affected colonic mucosa of patients with ITB in comparison to control biopsies

Fifty-one proteins were differentially expressed in biopsies from patients with ITB with reference to controls with the criteria of iTRAQ ratio <0.7 or >1.3, in at least one set of iTRAQ experiment. Twenty-three proteins were under-expressed in ITB and 28 proteins were overexpressed with respect to controls. Among those proteins, which were identified in more than one experiment, four proteins (myosin-11, macrophage migration inhibitory factor, heterogeneous nuclear ribonucleoprotein A1-like 2 and myosin-10) were significantly underexpressed (p value < 0.05) and four proteins (histone H2B type 1-B, hemoglobin subunit alpha, beta and delta) were overexpressed (p value < 0.05) in macroscopically affected colonic mucosa of patients with ITB with reference to control biopsies.

### Differential expression of proteins in the macroscopically affected colonic mucosa of patients with CD in comparison to control colonic biopsies

Seventy-eight proteins were differentially expressed in the colonic mucosa of patients with CD with reference to control biopsies with the criteria of ratio <0.7 or >1.3, in at least one set of iTRAQ experiment. Nineteen proteins were under-expressed and 59 proteins were overexpressed in patients with CD with respect to control biopsies. Among those proteins, which were identified in more than one experiment, six proteins were significantly underexpressed (alpha 1 acid glycoprotein, fatty acid-binding protein, serum albumin, IG kappa chain, mucin-2 precursor, and serotransferrin) (p value < 0.05) and 13 proteins were over-expressed (apolipoprotein A1, annexin A5, myosin-14, glyceraldehyde-3-phosphate dehydrogenase, histone H2B type 1-B, tropomyosin alpha-1 chain, transgelin, tropomyosin alpha-4 chain, L-lactate dehydrogenase B chain, fructose-bisphosphate aldolase A, peroxiredoxin-1, cofilin-1, protein disulfide-isomerase) (p value < 0.05) in patients with CD with reference to controls.

### Differential expression of proteins in macroscopically affected colonic mucosa of patients with CD and ITB

**Figure 3:**
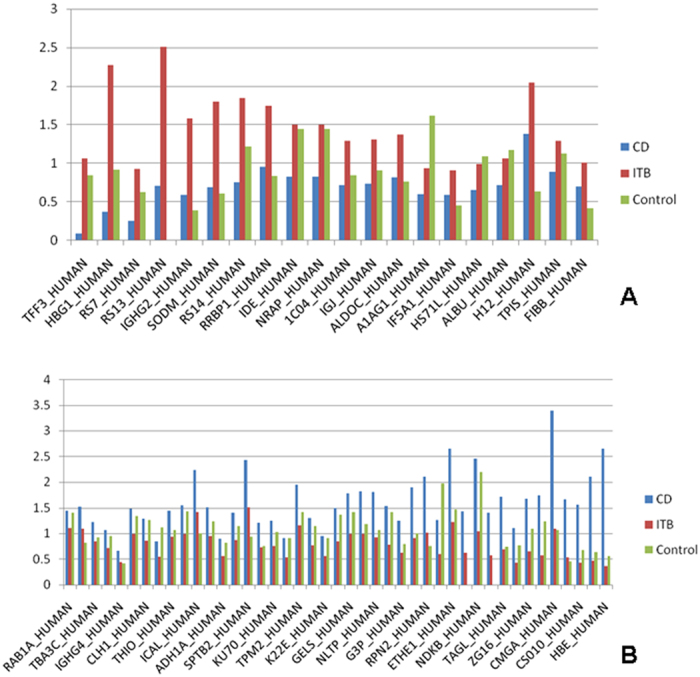
Bar charts showing the expression levels of the differentially expressed proteins in macroscopically affected colonic mucosa of patients with CD, ITB and control biopsies in the discovery phase. (**A**) Proteins underexpressed in patients with CD in comparison to that in those with ITB. (**B**) Proteins overexpressed in patients with CD in comparison to that in those with ITB.

Sixty-three proteins were differentially expressed in the colonic mucosa of patients with patients with CD and ITB in at least one set of iTRAQ experiment. Twenty proteins were underexpressed and 41 proteins were overexpressed in the colonic mucosa of patients with CD with respect to that in colonic mucosa of patients with ITB. Among those proteins, which were identified in more than one experiment, two proteins were significantly underexpressed (p value <0.05, ratio <0.7) and nine proteins were overexpressed (p value < 0.05, ratio > 1.3) in patients with CD with reference to ITB. List of all the proteins differentially expressed are shown in Tables 3 and 4 and Fig. 3.

### Bioinformatics analysis

**Figure 4:**
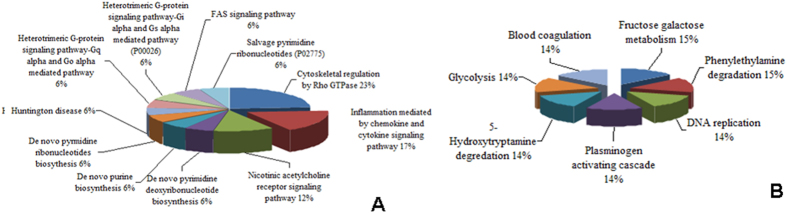
(**A**) Pie chart showing the Gene ontology annotation and the pathway distribution of underexpressed proteins in the macroscopically affected mucosa of patients with CD and ITB. **(B)** Pie chart showing the Gene ontology annotation and the pathway distribution of overexpressed proteins in the macroscopically affected mucosa of patients with CD and ITB.

A Bioinformatics analysis of both overexpressed and underexpressed proteins was carried out in PANTHER. The molecular function, biologic process and cellular location of these proteins was similar to the overall proteome covered. A pathway analysis of these differentially expressed proteins was carried out in PANTHER. The different pathways, with which these differentially expressed proteins possibly are involved, has been shown in Fig. 4. The underexpressed proteins were annotated to fructose galactose metabolism, apoptosis signaling pathway, plasminogen activating cascade, Parkinson’s disease, gonadotropin releasing hormone receptor pathway, glycolysis and blood coagulation pathways. On the other hand, the overexpressed proteins were annotated to pathways including cytoskeletal regulation, inflammation, integrin signaling pathway, hypoxia, oxidative stress response, FAS signaling pathway and G-protein signaling pathways.

### Validation of differentially expressed proteins using immunohistochemistry

**Figure 5:**
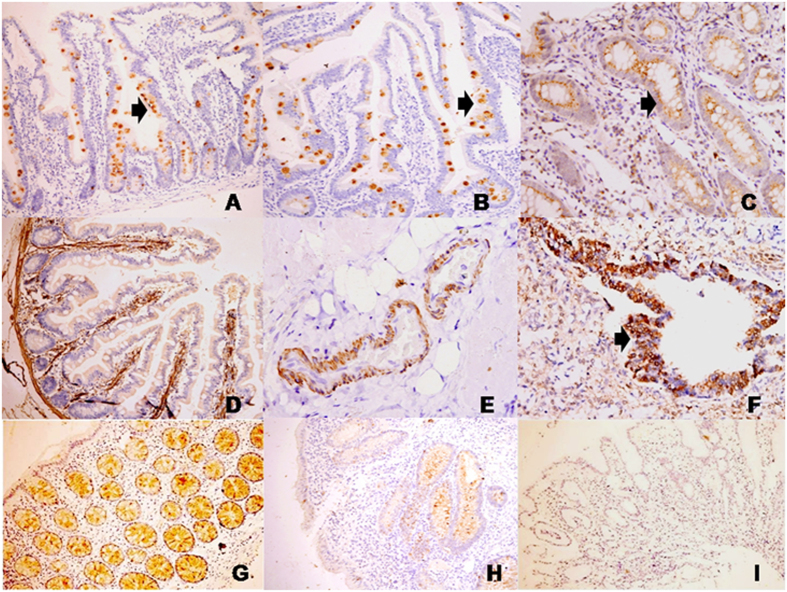
Microphotograph showing normal expression pattern of trefoil factor-3 [arrow] ((**A**), IHC [TFF3] x40), IgGFc-binding protein [arrow] ((**B**), IHC [FCGBP] x40), Myosin [arrow] ((**C**), IHC [MYH] x40), transgelin ((**D**), IHC [SM22] x40), tropomyosin ((**E**), IHC [Tropomyosin] x40) and Thioredoxin protein [arrow] ((**F**), IHC [TRX] x40). Comparative expression patterns of trefoil factor-3 in colon biopsies: (**G**) showing trefoil factor-3 expression score 12 ((**G**), IHC [TFF3] x40), expression score 6 ((**H**), IHC [TFF3] x40), and expression score 0 ((**I**), IHC [TFF3] x40).

Amongst the entire protein expression pattern studied by immunohistochemistry in the colonic biopsies, trefoil factor 3 expression pattern was significantly different in ITB in comparison to the biopsies from patients with CD in the initial analysis on 29 patients. While in the former the expression of trefoil factor 3 was predominantly weak to moderate, in ITB expression of trefoil factor 3 was always moderate to strong. However, this was not observed when we increased the sample size to 52 (Table 5). The expression patterns of IgGFc-binding protein, myosin, thioredoxin, transgelin and tropomysin proteins were not different in colonic biopsies in either of the diseases. Tropomysin was expressed in blood vessels and was not expressed in the epithelial cells. All other proteins as described above were however expressed over colonic epithelial cells. While trefoil factor 3 and IgGFc-binding protein proteins were expressed mostly in the goblet cell cytoplasm (Fig. 5A,B); Myosin and thioredoxin proteins were expressed in the epithelial cell cytoplasm in the control biopsies. Transgelin and topomyosin proteins were seen in the wall of the blood vessels (Fig. 5C–F). The expression of myosin, thioredoxin and transgelin proteins were particularly strong in both the CD and ITB in colonic biopsies.

## Discussion

The present study is the first ever proteomics analysis of mucosal biopsies from patients with CD and ITB and it has revealed several promising biomarker candidates for differentiation between these two diseases. In five sets of iTRAQ experiments, 533 proteins were identified and 241 proteins were quantified. Overall 63 proteins were differentially expressed in the colonic mucosa of patients with CD and ITB in at least one set of iTRAQ experiment, of them 11 proteins were differentially expressed in more than one set of experiments. Of them, We propose that trefoil factor 3, fatty acid synthase, Myosin 14, Myosin 11, Human thioredoxin 1, IgG Fc-binding protein, transgelin, and tropomyosin as potential candidates for biomarker development for differentiation between CD and ITB.

The proteins, which were differentially expressed in the colonic mucosa of patients with CD and ITB, may be playing a role in the pathogenesis of the disease or they may have appeared in the tissue as a consequence of thee diseases. For example, significantly lower expression of trefoil factor-3 level in patients with CD is likely to be marker of the activity of the disease. While we included both CD and ITB in the their active phase, the differentially lower expression of trefoil peptide-3 in patients with CD in comparison to that in ITB is reflective of larger area of mucosal involvement in those with CD. In one of our another study, we observed a higher level of serum trefoil factor-3 in the serum of patients with healed stage of ulcerative colitis in comparision to those having active disease^25^. Both these observations suggest that trefoil factor 3 may be a good marker of disease activity. Trefoil factor-3 is one of the three small, compact proteins expressed by the goblet cells in the gastrointestinal tract^26^. In addition to having anti-inflammatory properties, trefoil peptide also play an important role in the maintenance and repair of the gastrointestinal tract mucosa. The expression of trefoil is suppressed by tumor necrosis factor-α, the level of which remains high in patients with CD^27^.

While, distal and terminal ileum is involved both in CD and ITB, the quantum of mucosal involvement is more in patients with CD than that in ITB. A four-fold increase in the expression of fatty acid synthase in patients with CD as compared to that in ITB may partly be attributed to the higher altered fat metabolism and enterohepatic circulation of bile acids in patients with CD than that in ITB^28^,^29^,^30^.

While in the discovery experiments, several proteins were differentially expressed in the colonic mucosa of patients with CD and ITB with the ratio cut-off of <0.7 and >1.3; a very few proteins were expressed with sufficiently high fold differences required for development of a candidate biomarker. Whereas the individual pairs of the biopsies yielded several differentially expressed proteins in each experiment, this was not reflected consistently in all the five experiments. Furthermore, a lower level of differential expression of proteins in the colonic biopsies of patients with CD and ITB was also reflected by similar pattern of expression of these proteins by immunohistochemistry in a larger cohort of biopsies from patients with CD and ITB. This phenomenon may partly be attributed to the heterogeneity of the colonic mucosal biopsies as both CD and ITB are very diverse diseases. As colon contains four layers and the depth of involvement in both CD and ITB may vary in individual patients and thereby the tissue obtained from the edge of ulcers may also vary. These cell layers will have different protein expression profiles. The variable depth of biopsies therefore might have contributed to the heterogeneity of the proteome and skewed the quantitative proteome data. A proteome analysis in a more histologically directed manner is perhaps a better approach in this scenario. Since the intestine is a complex tissue, isolation and proteome analysis of a particular cell type involved actively in the disease process, such as colonocytes, is likely to provide a proteome enriched in low abundant proteins and a better resolution of proteome. Laser capture micro-dissection of intestinal biopsy specimens to obtain specific cells or density gradient separation to separate various cell types could be another strategy in this direction for future studies^31^. A complementary technique, MALDI imaging, may also be useful to obtain peptide signature for a particular layer of the intestine^32^.

In conclusion, a comparative analysis of the proteome profile of colonic biopsies from the macroscopically affected areas of the colons of patients with CD and ITB yielded 533 proteins of which 241 proteins could be quantified. There were differentially expressed proteins in the tissue proteome of patients with CD and ITB. Further experiments are required using a larger cohort of homogeneous tissue samples in order to find a biomarker for differentiation between CD and ITB.

## Additional Information

**How to cite this article**: Rukmangadachar, L. A. *et al*. Proteome analysis of the macroscopically affected colonic mucosa of Crohn's disease and intestinal tuberculosis. *Sci. Rep.* **6**, 23162; doi: 10.1038/srep23162 (2016).

## Figures and Tables

**Table 1:** iTRAQ study design.

**Set**	**114**	**115**	**116**	**117**
1	Ref Sample	CD1	ITB1	N1
2	Ref Sample	ITB2	N2	CD2
3	Ref Sample	N3	CD3	ITB3
4	Ref Sample	CD4	ITB4	N4
5	Ref Sample	ITB5	CD5	N5

CD 1-5: Patients with Crohn‘s’ disease; ITB 1-5: patients with Intestinal Tuberculosis; N1-5: Control biopsies Reference sample consisted of pool of all the samples.

**Table 2:** Details of the number of proteins identified in each set of iTRAQ experiment.

	**Set 1**	**Set 2**	**Set 3**	**Set 4**	**Set 5**	Total (nonredundant*)
Proteins identified	271	244	213	258	139	533
Proteins quantified	134	131	92	147	72	241
False discovery rate	5.32%	1.17%	0.65%	1.95%	1.47%	–

^*^139 proteins among these were identified and quantified in at least two sets of experiments.

**Table 3:** List of the underexpressed proteins in macroscopically affected colonic mucosa of patients with CD with respect to that in ITB.

SwissProtAccession No.	**Uniprot ID**	**Name**	**Gene Name**	**CD/ITB**
TFF3_HUMAN	Q07654	Trefoil factor 3	TFF3	0.08
HBG1_HUMAN	P69891	Hemoglobin subunit gamma-1	HBG1	0.16
RS7_HUMAN	P62081	40S ribosomal protein S7	RPS7	0.27
RS13_HUMAN	P62277	40S ribosomal protein S13	RPS13	0.28
IGHG2_HUMAN	P01859	Ig gamma-2 chain C region	IGHG2	0.37
SODM_HUMAN	P04179	Superoxide dismutase [Mn], mitochondrial	SOD2	0.38
RS14_HUMAN	P62263	40S ribosomal protein S14	RPS14	0.41
RRBP1_HUMAN	Q9P2E9	Ribosome-binding protein 1	RRBP1	0.55
IDE_HUMAN	P14735	Insulin-degrading enzyme	IDE	0.55
NRAP_HUMAN	Q86VF7	Nebulin-related-anchoring protein	NRAP	0.55
1C04_HUMAN	P30504	HLA class I histocompatibility antigen,Cw-4 alpha chain	HLA-C	0.55
IGJ_HUMAN	P01591	Immunoglobulin J chain	IGJ	0.56
ALDOC_HUMAN	P09972	Fructose-bisphosphate aldolase C	ALDOC	0.59
A1AG1_HUMAN	P02763	Alpha-1-acid glycoprotein 1	ORM1	0.64
IF5A1_HUMAN	P63241	Eukaryotic translation initiation factor 5A-1	EIF5A	0.65
HS71L_HUMAN	P34931	Heat shock 70 kDa protein 1-like	HSPA1L	0.66
ALBU_HUMAN	P02768	Serum albumin	ALB	0.68
H12_HUMAN	P16403	Histone H1.2	HIST1H1C	0.68
TPIS_HUMAN	P60174	Triosephosphate isomerase	TPI1	0.69
FIBB_HUMAN	P02675	Fibrinogen beta chain	FGB	0.69

**Table 4:** List of the overexpressed proteins in macroscopically affected colonic mucosa of patients with CD with respect to that in ITB.

**Protein**	**Uniprot ID**	**Name**	**Gene Name**	**CD/ITB**
RAB1A_HUMAN	P62820	Ras-related protein Rab-1A	RAB1A	1.31
ALDH2_HUMAN	P05091	Aldehyde dehydrogenase, mitochondrial	ALDH2	1.39
TBA3C_HUMAN	Q13748	Tubulin alpha-3C/D chain	TUBA3C	1.46
LUM_HUMAN	P51884	Lumican	LUM	1.48
IGHG4_HUMAN	P01861	Ig gamma-4 chain C region	IGHG4	1.49
VINC_HUMAN	P18206	Vinculin	VCL	1.49
CLH1_HUMAN	Q00610	Clathrin heavy chain 1	CLTC	1.50
FCGBP_HUMAN	Q9Y6R7	IgGFc-binding protein	FCGBP	1.54
THIO_HUMAN	P10599	Thioredoxin	TXN	1.55
MYLK_HUMAN	Q15746	Myosin light chain kinase, smooth muscle	MYLK	1.57
ICAL_HUMAN	P20810	Calpastatin	CAST	1.58
VDAC1_HUMAN	P21796	Voltage-dependent anion-selective channel protein 1	VDAC1	1.58
ADH1A_HUMAN	P07327	Alcohol dehydrogenase 1A	ADH1A	1.59
MYH14_HUMAN	Q7Z406	Myosin-14	MYH14	1.61
SPTB2_HUMAN	Q01082	Spectrin beta chain, non-erythrocytic 1	SPTBN1	1.62
ROAA_HUMAN	Q99729	Heterogeneous nuclear ribonucleoprotein A/B	HNRNPAB	1.65
KU70_HUMAN	P12956	X-ray repair cross-complementing protein 6	XRCC6	1.65
K2C6B_HUMAN	P04259	Keratin, type II cytoskeletal 6B	KRT6B	1.69
TPM2_HUMAN	P07951	Tropomyosin beta chain	TPM2	1.69
TBB5_HUMAN	P07437	Tubulin beta chain	TUBB	1.71
K22E_HUMAN	P35908	Keratin, type II cytoskeletal 2 epidermal	KRT2	1.71
MYH11_HUMAN	P35749	Myosin-11	MYH11	1.76
GELS_HUMAN	P06396	Gelsolin	GSN	1.82
TPM1_HUMAN	P09493	Tropomyosin alpha-1 chain	TPM1	1.84
NLTP_HUMAN	P22307	Non-specific lipid-transfer protein	SCP2	1.95
AGR3_HUMAN	Q8TD06	Anterior gradient protein 3 homolog	AGR3	1.96
G3P_HUMAN	P04406	Glyceraldehyde-3-phosphate dehydrogenase	GAPDH	2.02
RADI_HUMAN	P35241	Radixin	RDX	2.07
RPN2_HUMAN	P04844	Dolichyl-diphosphooligosaccharide–protein glycosyltransferase subunit 2	RPN2	2.08
1A01_HUMAN	P30443	HLA class I histocompatibility antigen, A-1 alpha chain	HLA-A	2.09
ETHE1_HUMAN	O95571	Protein ETHE1, mitochondrial	ETHE1	2.17
FIBA_HUMAN	P02671	Fibrinogen alpha chain	FGA	2.30
NDKB_HUMAN	P22392	Nucleoside diphosphate kinase B	NME2	2.36
A1AG2_HUMAN	P19652	Alpha-1-acid glycoprotein 2	ORM2	2.46
TAGL_HUMAN	Q01995	Transgelin	TAGLN	2.51
HNRDL_HUMAN	O14979	Heterogeneous nuclear ribonucleoprotein D-like	HNRPDL	2.57
ZG16_HUMAN	O60844	Zymogen granule membrane protein 16	ZG16	2.58
TBB6_HUMAN	Q9BUF5	Tubulin beta-6 chain	TUBB6	3.02
CMGA_HUMAN	P10645	Chromogranin-A	CHGA	3.10
LEG1_HUMAN	P09382	Galectin-1	LGALS1	3.11
CS010_HUMAN	Q969H8	UPF0556 protein C19orf10	C19orf10	3.63
FAS_HUMAN	P49327	Fatty acid synthase	FASN	4.50
HBE_HUMAN	P02100	Hemoglobin subunit epsilon	HBE1	7.22

**Table 5:** Protein expression pattern by Immunohistochemistry in colon biopsies.

	Weak(1–3)	Moderate(4-8)	Strong(9-12)	Fisher’s exactp value
Trefoil factor-3 (n-52)				
CD	7	13	9	0.25
ITB	10	6	7	
IgGFc-binding protein (n-27)				
CD	4	3	7	0.3
ITB	1	6	6	
Myosin (n-29)				
CD	0	2	12	0.9
ITB	0	2	13	
Thioredoxin (n-29)				
CD	0	0	14	1
ITB	0	0	15	
Trangelin (n-29)				
CD	0	0	14	1
ITB	0	0	15	
Tropomysin (n-29)				
CD	0	0	0	1
ITB	0	0	0	
